# Comparative genomics of *Sneathia* species: insights into pathogenicity and adaptation to the human niche

**DOI:** 10.1186/s12866-026-05201-6

**Published:** 2026-05-27

**Authors:** Paweł Łaniewski, Nicole R. Jimenez, Bonnie L. Hurwitz, Melissa M. Herbst-Kralovetz

**Affiliations:** 1 Department of Basic Medical Sciences, College of Medicine-Phoenix, University of Arizona, 425 N 5th Street, Phoenix, AZ 85004 USA; 2 Department of Obstetrics and Gynecology, College of Medicine-Phoenix, University of Arizona, Phoenix, AZ USA; 3 Bioinformatics Research Center and Department of Biological Sciences, College of Sciences, North Carolina State University, Raleigh, NC USA

**Keywords:** Anaerobe, Bacterial vaginosis, Cervical cancer, Cervical dysplasia, Chorioamnionitis, Dysbiosis, HPV, Preterm birth, Urethritis, Vaginal microbiome

## Abstract

**Background:**

*Sneathia* are urogenital anaerobes linked to bacterial vaginosis, preterm birth, chorioamnionitis, urethritis, and HPV-mediated cervical carcinogenesis. Two species have been identified: *Sneathia vaginalis* and *Sneathia sanguinegens*. Yet, due to their fastidious nature, our knowledge of their pathogenicity is limited.

**Methods:**

Comparative genomic analysis of 12 *Sneathia* genomes, including two newly sequenced via whole-genome sequencing, was used to evaluate the pathogenic potential of *S. vaginalis* and *S. sanguinegens*.

**Results:**

*Sneathia* species have small genomes (1.25–1.35 Mbp) with low (26.7–29.0%) GC content. The genomes of both species harbor a similar number of subsystems, mostly related to protein processing, metabolism, and energy. Both species encode subsystems for glycogen utilization and lactic and mixed acid fermentation. The genomes of *S. vaginalis* exhibit a higher potential for carbohydrate and carbohydrate derivative metabolism (gluconate and ascorbic acid catabolism, the Entner-Doudoroff pathway) providing a competitive advantage in nutrient-limited environments. *S. sanguinegens* genomes show a higher potential for amino sugar (hyaluronic acid and *N*-acetylneuraminic acid catabolism) and sulfur-containing amino acid metabolism. *Sneathia* species also encode unique subsystems related to stress response, including oxidative stress, heat shock, and antibiotic resistance. Finally, *Sneathia* genomes encode both core (shared by all strains) and accessory (specific for species or strains) virulence factors, including exotoxin, additional hemolysins, *O*-sialoglycoprotein endopeptidase, and adhesins.

**Conclusions:**

Both *S. vaginalis* and *S. sanguinegens* are highly adapted to the urogenital niche and exhibit high pathogenic capacities. Genomic information revealed potential mechanisms by which these pathogens enhance their competitiveness within the urogenital tract and likely facilitate microbe-microbe cooperation in polymicrobial communities.

**Supplementary Information:**

The online version contains supplementary material available at 10.1186/s12866-026-05201-6.

## Background

*Sneathia* is an emerging urogenital pathogen and a constituent of the dysbiotic vaginal microbiome [1]. Numerous clinical studies have shown that colonization of the vagina and cervix with these strictly anaerobic bacteria can cause serious adverse gynecologic, obstetric and reproductive sequelae, resulting in a significant impact on public health [1]. *Sneathia* has been associated with preterm birth [2, 3], cervicitis [4], pelvic inflammatory disease [5–7], infertility [6], as well as an increased risk of human papillomavirus (HPV) infections [8–10], development of cervical dysplasia and progression to cervical cancer [11–14]. In addition, *Sneathia* has been reported to cause maternal chorioamnionitis [15–19], postpartum bacteremia [20–22], neonatal meningitis [20], intrauterine fetal demise [23], septic abortion [24], and stillbirth [18]. *Sneathia* has also been detected in both women and men with urethritis [25]. Finally, *Sneathia* species also have been implicated in the pathogenesis of bacterial vaginosis (BV) [26], a common polymicrobial vaginal disorder affecting approximately one-third of reproductive-age women [27].

*Sneathia* is a genus of Gram-negative, non-pigmented, rod-shaped or pleomorphic, non-motile, non-spore-forming bacteria belonging to the family *Leptotrichiaceae* in the order Fusobacteriales. To date, two *Sneathia* species have been identified and properly validated *Sneathia vaginalis* [28] and *Sneathia sanguinegens* [29]. However, due to taxonomic reclassifications or redescriptions, previous reports might have used the names *Sneathia amnii* or *Leptotrichia amnionii* when referring to *S. vaginalis* [28]. Similarly, the name *Leptotrichia sanguinegens* was used in earlier reports to refer to *S. sanguinegens* [29]. Both species are fastidious, strictly anaerobic bacteria that require blood or serum, preferably of human source, for growth in vitro. Due to their complex nutritional requirements, limited *Sneathia* isolates have been cultured and sequenced. While approximately 40 *Sneathia* genomes are publicly available, only 10 were derived from isolate-based whole-genome sequencing (WGS). The remainder consist of metagenome-assembled genomes (MAGs), which are frequently fragmented and lack the integrity required for detailed comparative analysis. In this study, we sequenced two additional *Sneathia* strains via WGS and performed a comparative genomic analysis of 12 total WGS genomes (including 10 previously available sequences) to predict and compare the pathogenic potentials of the two *Sneathia* species. This study improves our understanding of how *S. vaginalis* and *S. sanguinegens* may drive pathophysiological changes in the urogenital microenvironment, contributing to adverse health outcomes.

## Methods

### Bacterial genomes, strains and growth conditions

All *Sneathia* genomes used in this study are listed in Table 1. After removing duplicates, a total of 10 publicly available genomes obtained via WGS were identified in the Bacterial and Viral Bioinformatics Resources Center (BV-BRC) platform. Additionally, two *Sneathia* isolates with unavailable genome sequences, *S. vaginalis* strain CCUG 64613 and *S. sanguinegens* strain CCUG 52978, were obtained from the Culture Collection University of Gothenburg (CCUG). Bacteria were cultured on brain heart infusion agar (Becton Dickinson) supplemented with 5% human serum, 1% yeast extract, 2% gelatin, 0.1% starch, 0.1% glucose at 37 °C under anaerobic conditions as previously described [30, 31]. Total genomic DNA was isolated from bacterial pellets using the DNeasy PowerSoil Pro Kit (Qiagen) and subjected to whole-genome sequencing (WGS).

**Table 1:** Bacterial genomes used in this study

Species	Strain	Source	Year	Country	Condition	GenBank
*Sneathia vaginalis*	SN35	Vagina	2011	USA	Preterm labor	CP011280
*Sneathia vaginalis*	CCUG 52976	Blood	2006	France	Peripartum bacteremia	JADBHK000000000
*Sneathia vaginalis*	CCUG 52977^ T^	Blood	2006	France	Peripartum bacteremia	JADBHL000000000
*Sneathia vaginalis*	CCUG 64370	Blood	2013	Sweden	Bacteremia	CP160094
*Sneathia vaginalis*	CCUG 64613*	Blood	2013	Sweden	Bacteremia	JBQVYR000000000
*Sneathia vaginalis*	WGS1539	*nr*	2021	Denmark	*nr*	CP170997
*Sneathia vaginalis*	WSU1	Vagina	2024	USA	BV	JBHMQL000000000
*Sneathia vaginalis*	WSU2	Vagina	2024	USA	BV	JBHMQK000000000
*Sneathia vaginalis*	WSU4	Vagina	2024	USA	BV	JBHLSP000000000
*Sneathia sanguinegens*	CCUG 41628^ T^	Blood	1999	Sweden	Complicated delivery	CP160095
*Sneathia sanguinegens*	CCUG 42621	Amniotic fluid	1999	Sweden	*nr*	JASSPP000000000
*Sneathia sanguinegens*	CCUG 52978*	Blood	2006	France	Peripartum bacteremia	JBQVYQ000000000

An asterisk indicates bacterial isolates that were sequenced in this study

* Abbreviations*: *BV* Bacterial vaginosis, *nr* not reported

### Whole-genome sequencing and genome assembly

The WGS was performed on extracted bacterial DNA at the University of Arizona PANDA Core for Genomics and Microbiome Research using PCR-Free Library Prep and the NextSeq1000 Platform with 300 cycle sequencing (Illumina). Trimmomatic was utilized to improve sequence read quality [32], which was evaluated with FastQC. Additionally, Kraken2 was used for human read filtration [33]. Genome assembly was performed with Unicycler release 16 [34], followed by quality checks with checkM2 [35] and Quast [36]. Genome annotation was performed using RAST tool kit (RASTk) [37]. The assembly quality was determined by measuring contiguity with the L50 metric, the number of contigs required to cover at least 50% of the assembly, and the N50 metric, the sequence length of the shortest contig at 50% of the total assembly length.

### Comparative genomic analysis

Comparative genomic analysis was performed using tools on the Bacterial and Viral Bioinformatics Resources Center (BV-BRC; https://www.bv-brc.org/): comprehensive genome analysis, bacterial genome tree, proteome comparison, and comparative systems [38]. A total of 12 *Sneathia* genomes (Table 1), including two genomes sequenced in this study and 10 other publicly available genomes obtained through WGS, were analyzed. A phylogenetic tree was generated on 100 single-copy orthologs using RAxML, multiple alignment using fast Fourier transform (MAFFT) alignment, and the default parameters. Protein sequence-based genome comparison was performed using bidirectional BLASTP. Comparative systems analysis was used to identify sets of proteins (or pathways) implemented in a specific biological process or structural complex. Protein family sorter analysis, which uses global protein families in the PATRIC (PAThosystems Resource Integration Center) resource (known as PGFams), was utilized for cross-genus comparisons. Average nucleotide identity (ANI) analysis was performed using FastANI [39] on the Enveomics platform [40].

### Statistical analyses

Statistical differences were determined by Mann–Whitney test using Prism 10 (GraphPad). *p* values below 0.05 were considered significant.

## Results

### Draft genomes of *Sneathia* isolates

**Fig. 1:**
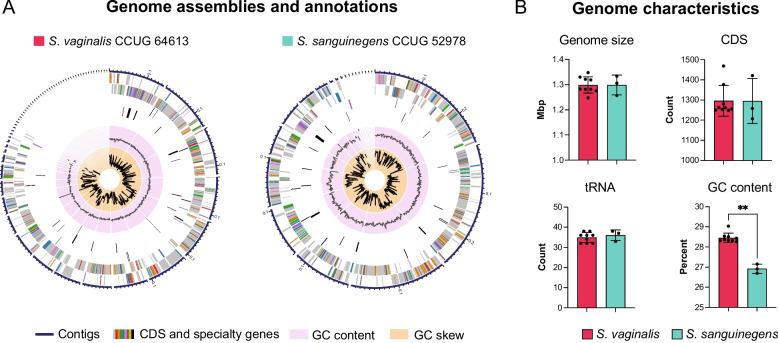
*Sneathia* species harbor relatively small genomes with a low number of coding sequences and low GC content. **A** Genome assemblies of two newly sequenced *Sneathia* strains. Circular graphical display of the genome annotations includes, from outer to inner rings, the contigs; CDS on the forward and reverse strands; RNA genes; antimicrobial resistance genes, genes encoding known virulence factors; GC content; and GC skew. **B** Comparison of genome characteristics between *S. vaginalis* and *S. sanguinegens* strains. Average size of genomes, number of CDS and number of genes encoding tRNA did not differ between the two *Sneathia* species. GC content was significantly (*p =* 0.0091) lower in *S. sanguinegens* compared to *S. vaginalis*. Abbreviations: CDS, coding DNA sequence

In order to perform comparative genomics analysis, we used 10 publicly available WGS *Sneathia* genomes (Table 1). In addition, we sequenced two additional *Sneathia* isolates obtained from the CCUG Collection (Gothenburg, Sweden): *S. vaginalis* strain CCUG 64613 and *S. sanguinegens* strain CCUG 52978. The draft genome of *S. vaginalis* strain CCUG 64613 comprised 65 contigs, with the total length of 1,318,768 bp and an average GC content of 28.26%, whereas the draft genome of *S. sanguinegens* strain CCUG 52978 comprised 22 contigs, with the total length of 1,256,673 bp and a GC content of 26.70% (Fig. 1A). Regarding assembly qualities, the *S. vaginalis* genome assembly had an L50 value of six and an N50 value of 105,948 bp, while the *S. sanguinegens* assembly had a more contiguous result with an L50 of three and a longer N50 of 198,861 bp. No plasmids were detected. Genome annotation of *S. vaginalis* CCUG 64613 revealed 1,249 coding DNA sequences (CDS) (of which 726 had functional assignment and 523 encoded hypothetical proteins), 34 tRNA genes, 24 repeat regions, and two rRNA genes. Genome annotation of *S. sanguinegens* strain CCUG 52978 revealed 1,207 CDS (including 709 proteins with functional assignment and 498 hypothetical proteins), 33 tRNA genes, 10 repeat regions, and two rRNA genes. Detailed genome assembly characteristics are included in Supplementary Table S1.

### Genome characteristics comparison

A total of 12 WGS genomes (nine *S. vaginalis* and three *S. sanguinegens*) were compared. All bacterial strains were isolated from human hosts between 1999 and 2024 and originated in Europe or the US (Table 1). Most strains were isolated from blood (six) or vagina (four); one strain was isolated from amniotic fluid. Regarding health conditions, three isolates were recovered from a patient with BV, three from patients with peripartum bacteremia, and one from a patient experiencing preterm labor. The comparison of genome characteristics showed that both *S. vaginalis* and *S. sanguinegens* harbor relatively small genomes, ranging from approximately 1.25 Mbp to 1.35 Mbp (Fig. 1B). There were no significant differences between species in the number of CDS (ranging from 1207 to 1469) or the number of tRNA genes (32 to 38). Genomes of both species exhibited relatively low GC content, yet on average the GC content was significantly (*p =* 0.0091) lower for *S. sanguinegens* (26.7–27.2%) compared to *S. vaginalis* (28.3–29.0%).

### Genetic relatedness

**Fig. 2:**
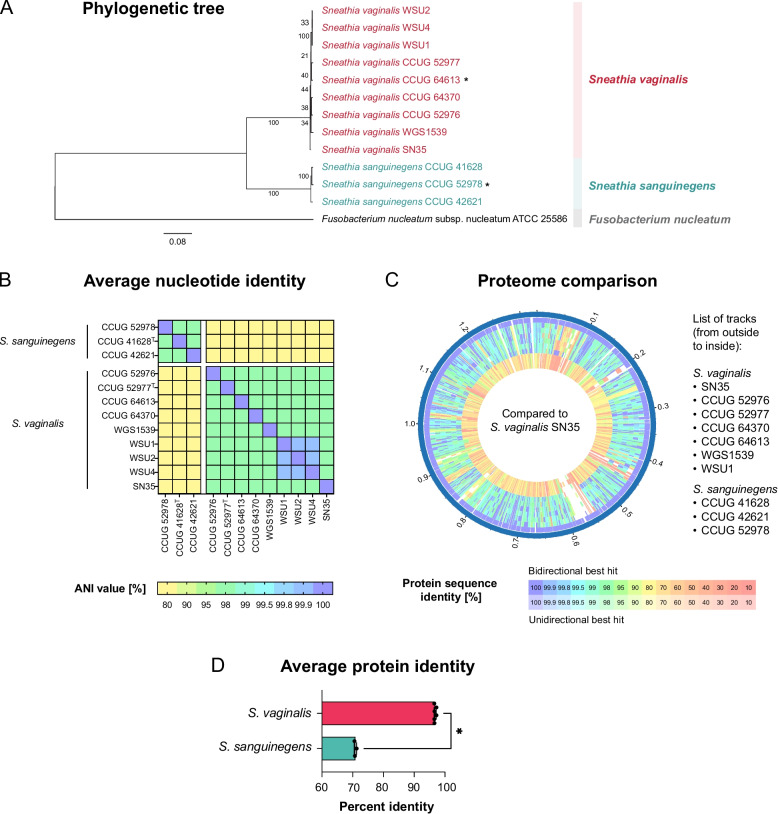
Phylogenetic analysis shows substantial evolutionary divergence between the two *Sneathia* species. **A** A phylogenetic tree revealed two distinct clusters representing *S. vaginalis* and *S. sanguinegens* species. The tree was generated using 100 single-copy orthologs and MAFFT alignment. The numbers above the tree branches indicate bootstrap values. A scale bar indicates the amount of evolutionary change represented by length of the branches. *Fusobacterium nucleatum* subsp. *nucleatum* was used as an outgroup. **B** An average nucleotide identities (ANI) analysis confirmed the taxonomic classification of newly sequenced strains (indicated by an asterisk) and other isolates. The heatmap shows a matrix of ANI values for all-to-all comparison of analyzed *Sneathia* genomes. ANI values > 80% and > 95% are indicative of genus and species classification, respectively. **C** Protein sequence-based comparison of *Sneathia* genomes. The circular plot shows protein alignments of the best BLAST hits for each genome when compared to the reference genome (*S. vaginalis* SN35). Hits are colored based on the protein sequence identity. **D** *S. vaginalis* strains displayed high protein identity (average 96.7%) with other *S. vaginalis* strains and lower protein identity (average 70.9%) with *S. sanguinegens* strains, further indicating their evolutionary relatedness

A phylogenetic analysis of 12 WGS genomes utilizing 100 single-copy orthologs (Supplementary Table S2) revealed two distinct clusters, each representing a *Sneathia* species, which was supported by 100% bootstrap values (Fig. 2A). All *Sneathia* members were clearly separated from *Fusobacterium nucleatum* utilized as an outgroup. Furthermore, average nucleotide identity (ANI) analysis of the *Sneathia* genomes showed a minimal identity of 80.8%, confirming relatedness at the genus levels (> 80%) (Fig. 2B). The analysis identified two distinct clusters corresponding to *S. sanguinegens* and *S. vaginalis*. Intraspecies ANI values ranged from 98.1% to 99.9%, exceeding the 95% threshold and supporting their current species classification. The phylogenetic positions confirmed the taxonomic classification of newly sequenced strains and other isolates. To measure evolutionary distance among *Sneathia* members, we further compared the protein sequences encoded by *Sneathia* genomes against the reference *S. vaginalis* SN35 (Fig. 2C). *S. vaginalis* strains exhibited, on average, 96.7% protein identity, whereas *S. sanguinegens* strains displayed 70.9% protein identity (Fig. 2D). This is consistent with their taxonomy as different species within the same genus.

### Comparative systems

**Fig. 3:**
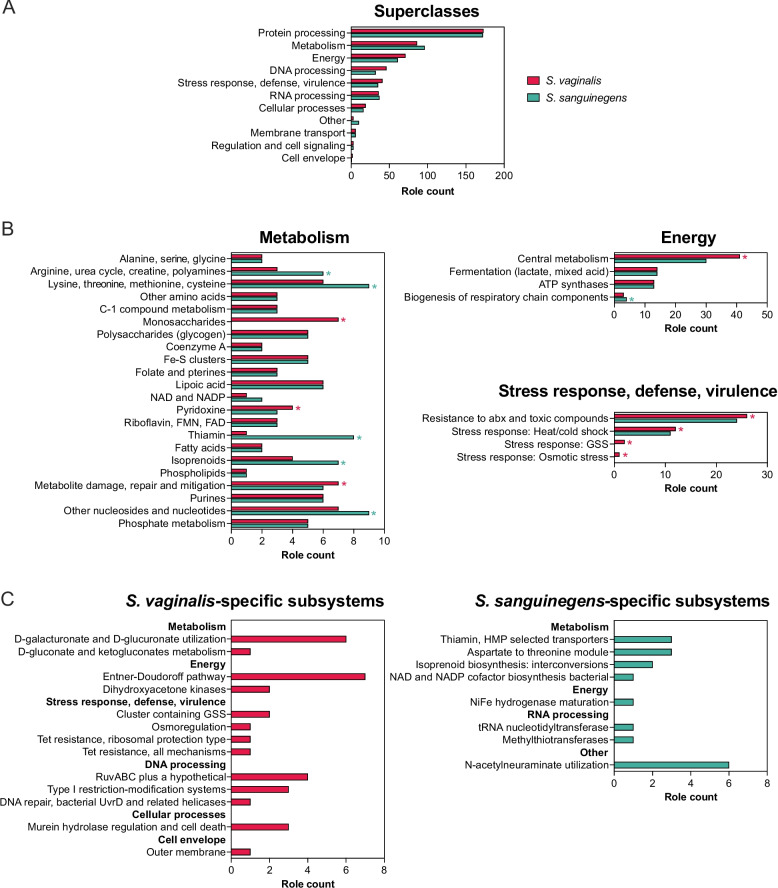
The genomes of *S. vaginalis* exhibit a higher potential for carbohydrate metabolism and stress responses, while those of *S. sanguinegens* show a higher potential for amino acid, amino sugar and vitamin metabolism. **A** Functionally, all *Sneathia* genomes harbored many subsystems involved in protein processing, metabolism, and energy. **B** The *Sneathia* species differed in subsystems belonging primarily to the metabolism, energy, and stress response, defense and virulence superclasses. **C** Unique subsystems for *S. vaginalis* were related to metabolism, energy, stress responses DNA processing, cellular processes, and cell envelope, whereas unique subsystems for *S. sanguinegens* were related to metabolism, energy, RNA processing, and *N*-acetylneuraminate (sialic acid) utilization. The bar charts illustrate the number of unique functional roles assigned at the superclass (**A**), subclass (**B**) or subsystem level (**C**). An asterisk indicates an enrichment of functional roles in *S. vaginalis* (red) or *S. sanguinegens* (green). Abbreviations: ATP, adenosine triphosphate; FAD, flavin adenine dinucleotide; FMN, flavin mononucleotide; GSS, glutathione synthetase; HMP, hydroxymethylpyrimidine; NAD, nicotinamide adenine dinucleotide; NADP, NAD phosphate; Tet, tetracycline

The comparative systems analysis was performed for each *Sneathia* species (Supplementary Tables S3 and S4) to identify a set of functions implemented in a specific biological process or a structural complex that can be generalized as a pathway. At the superclass level, both *S. vaginalis* and *S. sanguinegens* genomes harbored similar numbers of subsystems and unique functional roles (Fig. 3A). These were asserted mostly to protein processing (173 and 172, respectively), metabolism (86 and 96) and energy (71 and 61), followed by DNA processing (46 and 32), stress response, defense, and virulence (41 and 35), and RNA processing (36 and 37) (Supplementary Table S5). Yet, at the subclass level, there were differences in the number of functional roles between *Sneathia* species, mostly related to metabolism, energy, and stress responses (Fig. 3B). Regarding metabolism, *S. vaginalis* genomes harbored unique roles for monosaccharides metabolism (specifically D-galacturonate and D-glucuronate utilization and D-gluconate and ketogluconates metabolism); and additional roles in metabolite damage and repair (Nudix hydrolases) and vitamin/cofactor (pyridoxin) subsystems (Fig. 3C). Both *Sneathia* species contained functions related to polysaccharides, such as glycogen metabolism. Conversely, *S. sanguinegens* genomes encoded additional roles related to amino acid metabolism (polyamine metabolism; aspartate to threonine module), vitamin/cofactor metabolism (thiamin transport, salvage and uptake; NAD and NADP biosynthesis), lipids (isoprenoid biosynthesis; mevalonate metabolic pathway) and nucleotides (adenosyl nucleosides). To produce energy, both species may utilize fermentation (lactic acid or mixed acid), whereas only *S. vaginalis* harbored genes encoding dihydroxyacetone kinases and enzymes involved in the Entner-Duodoroff pathway. *S. sanguinegens* harbored a unique role for NiFe hydrogenase maturation, which is involved in the biogenesis of respiratory chain components. Regarding stress responses, defense, and virulence, *S. vaginalis* genomes contained unique functional roles related to osmotic stress, glutathione synthesis, tetracycline resistance, and an additional role for heat shock responses. Finally, *S. vaginalis* harbored unique subsystems for DNA processing, cellular processes, and cell envelope, whereas *S. sanguinegens* harbored unique subsystems involved in RNA processing and utilization of amino sugars and nucleotide sugars, specifically *N*-acetylneuraminic acid (sialic acid).

### Protein family functions

**Fig. 4:**
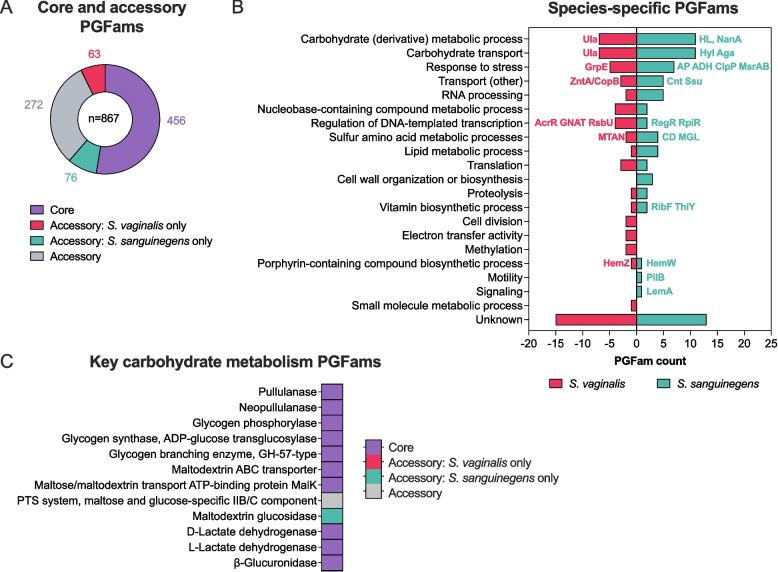
*Sneathia* genomes encode species-specific protein families related primarily to carbohydrate metabolism, stress responses, and transport. **A** A total of 867 global protein families (PGFams, as defined in the PATRIC database) were identified. The protein family sorter analysis revealed 456 core PGFams (shared among all 12 *Sneathia* genomes) and 411 accessory families, including 63 unique to *S. vaginalis* and 76 unique to *S. sanguinegens*. **B** The bar charts illustrate PGFams specific for *S. vaginalis* or *S. sanguinegens*, grouped by biological processes [Gene Ontology (GO) annotations]. Unique protein families were involved mostly in carbohydrate and carbohydrate derivative metabolic processes, carbohydrate transport, or responses to stress. Key proteins with a potential impact on *Sneathia* pathogenicity are listed adjacent to the bars. **C** The analysis also revealed that all *Sneathia* strains, regardless of species, harbored enzymes and proteins related to glycogen breakdown and synthesis, maltodextrin transport, lactic acid fermentation, and glucuronide deconjugation

The protein family sorter analysis was performed to examine the distribution of specific protein families (PGFams) across *Sneathia* genomes. A total of 867 protein families were identified (Supplementary Table S6). The analysis revealed 456 PGFams (52.6%) to be shared among all tested 12 *Sneathia* genomes, referred to as the core genome, and 411 PGFams (47.4%) to be present in a subset of genomes, referred to as the accessory genome (Fig. 4A). Within the accessory families, 63 PGFams were present in all *S. vaginalis* genomes but absent in *S. sanguinegens*, whereas 76 PGFams were uniquely present in *S. sanguinegens* genomes and absent in *S. vaginalis* (Supplementary Table S7 and S8). The other 272 accessory protein families were distributed in various *Sneathia* strains, independent of species designation.

When grouped based on biological processes using Gene Ontology (GO) annotations, *S. vaginalis* and *S. sanguinegens* genomes encoded many species-specific proteins playing roles in carbohydrate or carbohydrate derivative metabolic processes and carbohydrate transport (Fig. 4B). Specifically, *S. vaginalis* encoded proteins involved in the utilization of L-ascorbic acid (Ula proteins) and phosphorylated β-glucosides as a carbon source. On the other hand, *S. sanguinegens* harbored genes encoding hyaluronate lyase (hyaluronidase, HL) precursor, *N*-acetylneuraminate lyase (sialic acid aldolase, NPL, NanA) and hyaluronate and *N*-acetylgalactosamine phosphotransferase systems. These enzymes and transporters are needed for catabolism of hyaluronic acid and amino sugars, which are key components of extracellular matrix and mucin glycoproteins. In addition, appropriate transcription regulators—specifically the repressor of hyaluronate and KDG utilization (RegR) and the sialic acid utilization regulator (RpiR family)—were found in *S. sanguinegens* genomes. These regulators were absent in *S. vaginalis*; however, other proteins regulating transcription were exclusively present in *S. vaginalis* (AcrR family, GNAT family, RsbU, MraZ). *Sneathia* genomes also harbored species-specific proteins that mediate stress responses, including those involved in heat shock, protein unfolding (GrpE and ClpP protease), and DNA damage/repair. In addition, *S. sanguinegens* harbored unique proteins involved in the response to anoxia and phosphate starvation (acid phosphatase, AP) and oxidative stress (alcohol dehydrogenase, ADH; methionine sulfoxide reductases, MsrAB). Other enzymes involved in cysteine/methionine catabolism (cysteine desulfurase, CD; methionine γ-lyase, MGL) or methionine salvage were present only in *S. sanguinegens* genomes. However, methylthioadenosine deaminase (MTAN), also crucial for recycling sulfur-containing metabolites, was exclusive to *S. vaginalis*. Furthermore, *Sneathia* species had unique proteins for heme acquisition, riboflavin and thiamin metabolism, and transport of metal ions. Finally, only *S. sanguinegens* harbored genes encoding proteins binding and transporting alkanesulfonates (Ssu), a type IV fimbrial assembly ATPase (PilB), and a kinase of the two-component signal transduction system (LemB).

Since glycogen is an abundant polysaccharide in the lower female reproductive tract, we investigated key enzymes related to complex carbohydrate utilization among *Sneathia* genomes (Fig. 4C). All *Sneathia* genomes, independent of strain or species, harbored genes encoding pullulanase, neopullulanase, and glycogen phosphorylase, which facilitate the breakdown of glycogen to maltodextrins and shorter oligosaccharides. Furthermore, all genomes encoded systems that transport maltodextrins, yet presence of maltose transporters was more strain specific. Intriguingly, all *Sneathia* genomes also possessed enzymes involved in glycogen synthesis, such as glycogen synthase and glycogen branching enzyme. Genes encoding D-lactate and L-lactate dehydrogenases were also present in all genomes, showing the capability of *Sneathia* species to produce energy via lactic acid fermentation. Finally, all *Sneathia* also encoded β-glucuronidase, a key enzyme involved in deconjugation of glucuronides, which can lead to the reactivation of toxins, drugs, and hormones.

### Virulence factors

**Fig. 5:**
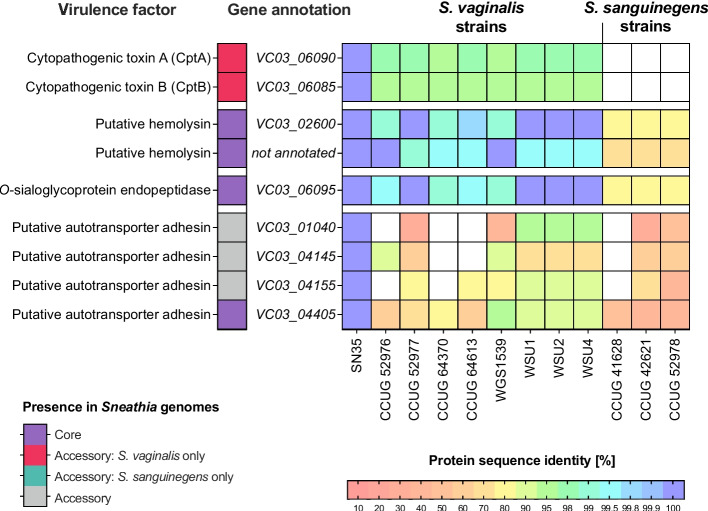
*Sneathia* genomes encode both core (shared by all strains) and accessory (specific for species or strains) virulence factors. The comparative proteome analysis revealed that the previously characterized cytopathogenic toxin A (CptA) and its transporter (CptB) are present in all *S. vaginalis* strains but absent in *S. sanguinegens* strains. Two putative hemolysins, a protease (*O*-sialoglycoprotein endopeptidase), and one putative autotransporter adhesin were identified as a core set (present in all tested *Sneathia* strains). The presence of three additional identified adhesins was strain-specific (indicated as accessory factors). The gene annotations refer to the *S. vaginalis* SN35 genome. The accompanying heatmap on the left indicates the protein sequence identity relative to *S. vaginalis* strain SN35. White squares indicate the absence of a protein in the corresponding strain

The virulence factors of *Sneathia* species are not well defined. To date, the most characterized protein is cytopathogenic toxin A (CptA). The genes encoding this toxin and its transporter (CptB) were uniquely harbored by all tested *S. vaginalis* strains and absent in *S. sanguinegens* strains (Fig. 5). Comparing these orthologs to *S. vaginalis* strain SN35, we found high sequence identity, measuring 97%–99% for CptA and 96%–98% for CptB (Supplementary Table S9). This high level of conservation indicates a critical function of these proteins. Besides CptA, we found two additional proteins with putative hemolysin activity. In contrast to CptA, these highly conserved hemolysins were present in both *S. vaginalis* (99%–100% identity) and *S. sanguinegens* strains (70%–82% identity). Moreover, *O*-sialoglycoprotein endopeptidase, which plays a role in cleaving sialyloglycoproteins and degradation of mucus, was also present in both *S. vaginalis* and *S. sanguinegens* strains. Finally, we found four putative autotransporter adhesins; of these, only one was present in all tested *S. vaginalis* and *S. sanguinegens* strains. The sequence identities of putative adhesins were also lower (39% to 97%) compared to other mentioned virulence factors.

## Discussion

The comparative genomic analysis of two *Sneathia* species revealed that these emerging urogenital pathogens are highly adapted to the human niche. Overall, the relatively small genome size, low number of genes, and low GC content indicates evolutionary adaptation to a specific microenvironment and high reliance on the host for nutrients and other chemical and physical resources. The genomes of *Sneathia* spp. (1.25–1.35 Mbp) are among the smallest in the *Leptotrichiaceae* family, ranking just above the closely related *Caviibacter abscessus* (1.22 Mbp) [41]. Since *Sneathia* species colonize the human urogenital tract as a part of dysbiotic polymicrobial consortia, the reduced genome size may also reflect reliance on the other community members and could further explain their fastidious nature.

Functional analysis identified some core and unique catabolic potentials of *Sneathia* species, particularly regarding carbohydrate metabolism. In the vagina, the most ubiquitous carbohydrate is glycogen, a complex glucose polymer released by exfoliated epithelial cells. In the optimal state, the vaginal microbiota is dominated by lactobacilli, which utilize glycogen byproducts as the main energy source via lactic acid fermentation, resulting in acidification of the mucosa. Yet, BV-associated bacteria, such as *Gardnerella* and *Prevotella*, also produce glycogen-degrading enzymes [42]. Similarly, *S. vaginalis* and *S. sanguinegens* genomes encode pullulanases and neopullulanses (glycogen-debranching enzymes) and maltodextrin and maltose transporters, suggesting that both species can effectively utilize glycogen as carbon and energy source. Although both *Sneathia* species harbor subsystems for lactic acid fermentation and mixed acid fermentation, *Sneathia* likely catabolize sugars through the latter process. This metabolic activity was previously evidenced for *S. sanguinegens* by the in vitro production of lactic acid and short-chain fatty acids (SCFAs), including formic and acetic acid [20, 29]. Notably, SCFAs have been identified as a metabolic hallmark of BV [43] and preterm birth [44] and have been shown to decrease vaginal epithelial barrier integrity, induce proinflammatory responses, and trophoblast function in vitro [44, 45].

*Sneathia* species also exhibited species-specific catabolic processes related to carbohydrate metabolism, including gluconate and L-ascorbic acid. Only *S. vaginalis* had subsystems for the Entner-Doudoroff pathway and genes encoding Ula proteins, which provide routes to catabolize/ferment gluconate or ascorbic acid, respectively. These unique pathways might provide *S. vaginalis* a competitive advantage in environments with limited nutrient availability and/or during sudden changes in nutrients (e.g., when ascending to the upper reproductive tract). Moreover, they might also play a role in *S. vaginalis’* growth, survival, and pathogenicity by regulating stress responses, adhesion, and production of virulence factors [46, 47].

On the other hand, *S. sanguinegens* genomes contained genes encoding enzymes for the degradation of mucus (*N*-acetylneuraminate lyase/sialidase) and extracellular matrix (hyaluronidase), features that were absent in *S. vaginalis* genomes. Both mucins and hyaluronic acid form physical barriers in the epithelium; thus *S. sanguinegens’* enzymatic degradation of these components can facilitate entry into epithelial tissue for nutrient acquisition and potential systemic spread. Furthermore, this breach in the mucosal barrier increases host susceptibility to sexually transmitted infections, including HPV [8–10]. Mucins are also the main component of the cervical plug, a structure formed during pregnancy to protect the fetus from ascending infections. *S. sanguinegens*’ disruption of the cervical barrier allows this organism and other bacteria within the polymicrobial community to ascend into the upper reproductive tract and to the fetus, potentially leading to severe adverse outcomes, such as chorioamnionitis [15–19], fetal demise [23], and septic abortion [24]. However, it is important to note that both *S. vaginalis* and *S. sanguinegens* possess the gene encoding *O*-sialoglycoprotein endopeptidase, in addition to unique proteolytic enzymes. These enzymatic profiles highlight potential cooperation among these two *Sneathia* species and other bacteria, in the degradation of mucins and other complex glycoconjugates. Notably, a previous study showed that *S. vaginalis* and *S. sanguinegens* often co-occur in the vagina [30].

Microorganisms residing in the human host encounter multiple stresses, including physical, chemical, and immune/biological. Consequently, these microbes have evolved mechanisms to cope with these stressors. Both *Sneathia* species harbored subsystems mediating resistance or tolerance to multiple types of antibiotics and metals (cadmium, copper), yet only *S. sanguinegens* possessed subsystems for tetracycline resistance. Oxidative stress, in particular, is a major challenge for anaerobic bacteria. *Sneathia* species had unique proteins for synthesis of glutathione, which mediates the redox state and protects bacteria by scavenging reactive oxygen species. Conversely, *S. sanguinegens* encoded MsrAB proteins, which repair oxidized methionine in proteins; these proteins are known in other human pathogens to be crucial for colonization, biofilm formation, and host response modulation [48].

Methionine and cysteine are critical for antioxidant defense by maintaining redox homeostasis and contributing to the synthesis of antioxidant molecules. Thus, the ability to synthesize or scavenge these sulfur-containing compounds is essential for bacterial survival and growth. Intriguingly, *S. sanguinegens* but not *S. vaginalis* genomes encoded cysteine desulfurase (CD) and methionine γ-lyase (MGL). These enzymes function, respectively, by forming iron-sulfur clusters in proteins and by allowing utilization of methionine as a carbon source, and they have also been implicated in bacterial pathogenesis. CD activity results in hydrogen sulfide production, which can damage host tissue and cause hemolysis [49], whereas MGL activity produces methanethiol and ammonia, a process implicated in causing malodor by periodontal bacteria [50]. The presence of genes encoding these key enzymes is likely to confer similar virulence advantages to *S. sanguinegens*.

Several vaginal bacteria are known to produce toxins (vaginolysin by *Gardnerella* species, inerolysin by *L. iners*), which enhance their competitiveness in the human niche. *S. vaginalis* also produces a large (226 kDa) pore-forming exotoxin [51]. This protein, named CptA, is the effector/passenger protein of a type Vb (two-partner) secretion system and requires the outer membrane transporter, CptB, to be effectively secreted. CptA has been shown to exhibit cytopathogenic activity against fetal membranes and hemolytic activity against human erythrocytes [51]. Notably, the *cptAB* genes were absent in *S. sanguinegens* genomes, yet *S. sanguinegens* colonies display β-hemolysis when grown on human blood agar [20]. Other toxins might be responsible for this activity, as two putative hemolysins (48 kDa and 32 kDa) were found across all analyzed *Sneathia* genomes. However, the abolished hemolytic activity observed in the *cptA* mutant [52] suggests that the specificity of these hemolysins may vary by strain. Finally, the presence of various autotransporter adhesins with relatively low protein identity between homologs suggests a strategy of immune evasion for *Sneathia* species and their potential adaptation to multiple niches within the host.

## Conclusions

The comparative genomic analyses revealed that the two *Sneathia* species have high pathogenic potential. While we identified some core functions common for both *S. vaginalis* and *S. sanguinegens*, we also identified species-specific pathways and virulence factors. These revealed potential mechanisms by which the *Sneathia* species enhance their competitiveness and survival within the urogenital tract and also facilitate cooperation between bacteria in polymicrobial communities. Our findings provide a foundation for future research into specific gene functions. Further studies should aim to validate gene expression and virulence factor production to better elucidate the functional impact of these proteins. Moreover, the utilization of human organotypic or animal models will be critical to comprehensively understand the pathogenesis of these emerging microorganisms within the human host.

## Supplementary Information

Supplementary Material 1. Table S1-S9.

## Data Availability

The draft genome assemblies for *S. vaginalis* strain CCUG 64613 and *S. sanguinegens* strain CCUG 52978 have been deposited in the Sequence Read Archive (SRA) under BioProject PRJNA1311619. Following SRA guidelines, contigs shorter than 200 bp were filtered out. The data are available under BioSample IDs SAMN50835186 and SAMN50835187, with raw sequencing reads accessible via accession numbers SRR36990655 and SRR36990656. Additional assembly information and parameters utilized can be found at https://github.com/hurwitzlab/vaginal_genome_assembly.

## Abbreviations

ADH: Alcohol dehydrogenase
ANI: Average nucleotide identity
ATP: Adenosine triphosphate
BEI: Biodefense and Emerging Infections Research Resources Repository
BLASTP: Protein basic local alignment search tool
BV: Bacterial vaginosis
BV-BRC: Bacterial and Viral Bioinformatics Resources Center
CCUG: Culture Collection University of Gothenburg
CD: Cysteine desulfurase
CDS: Coding DNA sequence
Cpt: Cytopathogenic toxin
FAD: Flavin adenine dinucleotide
FMN: Flavin mononucleotide
GO: Gene ontology
GSS: Glutathione synthetase
HL: Hyaluronate lyase
HMP: Hydroxymethylpyrimidine
HPV: Human papillomavirus
kDa: Kilodalton
MAFFT: Multiple alignment using fast Fourier transform
Mbp: Megabase pair
MGL: Methionine γ-lyase
MTAN: Methylthioadenosine deaminase
NAD: Nicotinamide adenine dinucleotide
NADP: NAD phosphate
NPL: *N*-Acetylneuraminate pyruvate lyase
PATRIC: PAThosystems Resource Integration Center
SCFA: Short-chain fatty acid
Tet: Tetracycline
WGS: Whole-genome sequencing

## References

[1] Theis KR, Florova V, Romero R, Borisov AB, Winters AD, Galaz J, et al. *Sneathia*: an emerging pathogen in female reproductive disease and adverse perinatal outcomes. Crit Rev Microbiol. 2021;47(4):517–42. 33823747 10.1080/1040841X.2021.1905606 PMC8672320

[2] Fettweis JM, Serrano MG, Brooks JP, Edwards DJ, Girerd PH, Parikh HI, et al. The vaginal microbiome and preterm birth. Nat Med. 2019;25(6):1012–21. 31142849 10.1038/s41591-019-0450-2 PMC6750801

[3] Brown RG, Marchesi JR, Lee YS, Smith A, Lehne B, Kindinger LM, et al. Vaginal dysbiosis increases risk of preterm fetal membrane rupture, neonatal sepsis and is exacerbated by erythromycin. BMC Med. 2018;16(1):9. 29361936 10.1186/s12916-017-0999-x PMC5782380

[4] Schwebke JR, Weiss HL. Interrelationships of bacterial vaginosis and cervical inflammation. Sex Transm Dis. 2002;29(1):59–64. 11773880 10.1097/00007435-200201000-00010

[5] Gondwe T, Ness R, Totten PA, Astete S, Tang G, Gold MA, et al. Novel bacterial vaginosis-associated organisms mediate the relationship between vaginal douching and pelvic inflammatory disease. Sex Transm Infect. 2020;96(6):439–44. 31810995 10.1136/sextrans-2019-054191 PMC7476288

[6] Haggerty CL, Totten PA, Tang G, Astete SG, Ferris MJ, Norori J, et al. Identification of novel microbes associated with pelvic inflammatory disease and infertility. Sex Transm Infect. 2016;92(6):441–6. 26825087 10.1136/sextrans-2015-052285 PMC5013099

[7] Mitchell CM, Haick A, Nkwopara E, Garcia R, Rendi M, Agnew K, et al. Colonization of the upper genital tract by vaginal bacterial species in nonpregnant women. Am J Obstet Gynecol. 2015;212(5):611 e611-619. 10.1016/j.ajog.2014.11.043 PMC4754962 25524398

[8] Lee JE, Lee S, Lee H, Song YM, Lee K, Han MJ, et al. Association of the vaginal microbiota with human papillomavirus infection in a Korean twin cohort. PLoS One. 2013;8(5):e63514. 23717441 10.1371/journal.pone.0063514 PMC3661536

[9] Onywera H, Williamson AL, Mbulawa ZZA, Coetzee D, Meiring TL. The cervical microbiota in reproductive-age South African women with and without human papillomavirus infection. Papillomavirus Res. 2019;7:154–63. 30986570 10.1016/j.pvr.2019.04.006 PMC6475661

[10] Cheng L, Norenhag J, Hu YOO, Brusselaers N, Fransson E, Ahrlund-Richter A, et al. Vaginal microbiota and human papillomavirus infection among young Swedish women. NPJ Biofilms Microbiomes. 2020;6(1):39. 33046723 10.1038/s41522-020-00146-8 PMC7552401

[11] Łaniewski P, Barnes D, Goulder A, Cui H, Roe DJ, Chase DM, et al. Linking cervicovaginal immune signatures, HPV and microbiota composition in cervical carcinogenesis in non-Hispanic and Hispanic women. Sci Rep. 2018;8(1):7593. 29765068 10.1038/s41598-018-25879-7 PMC5954126

[12] Audirac-Chalifour A, Torres-Poveda K, Bahena-Roman M, Tellez-Sosa J, Martinez-Barnetche J, Cortina-Ceballos B, et al. Cervical microbiome and cytokine profile at various stages of cervical cancer: a pilot study. PLoS One. 2016;11(4):e0153274. 27115350 10.1371/journal.pone.0153274 PMC4846060

[13] Mitra A, MacIntyre DA, Lee YS, Smith A, Marchesi JR, Lehne B, et al. Cervical intraepithelial neoplasia disease progression is associated with increased vaginal microbiome diversity. Sci Rep. 2015;5:16865. 26574055 10.1038/srep16865 PMC4648063

[14] Mancilla V, Jimenez NR, Bishop NS, Flores M, Herbst-Kralovetz MM. The vaginal microbiota, human papillomavirus infection, and cervical carcinogenesis: a systematic review in the Latina population. J Epidemiol Glob Health. 2024;14(2):480–97. 10.1007/s44197-024-00201-z PMC11176136 38407720

[15] Doyle RM, Harris K, Kamiza S, Harjunmaa U, Ashorn U, Nkhoma M, et al. Bacterial communities found in placental tissues are associated with severe chorioamnionitis and adverse birth outcomes. PLoS One. 2017;12(7):e0180167. 28700642 10.1371/journal.pone.0180167 PMC5507499

[16] Han YW, Shen T, Chung P, Buhimschi IA, Buhimschi CS. Uncultivated bacteria as etiologic agents of intra-amniotic inflammation leading to preterm birth. J Clin Microbiol. 2009;47(1):38–47. 18971361 10.1128/JCM.01206-08 PMC2620857

[17] Lannon SMR, Adams Waldorf KM, Fiedler T, Kapur RP, Agnew K, Rajagopal L, et al. Parallel detection of *Lactobacillus* and bacterial vaginosis-associated bacterial DNA in the chorioamnion and vagina of pregnant women at term. J Matern Fetal Neonatal Med. 2019;32(16):2702–10. 29478370 10.1080/14767058.2018.1446208 PMC6135717

[18] Vitorino P, Varo R, Castillo P, Hurtado JC, Fernandes F, Valente AM, et al. *Sneathia amnii* and maternal chorioamnionitis and stillbirth, Mozambique. Emerg Infect Dis. 2019;25(8):1614–6. 31310211 10.3201/eid2508.190526 PMC6649333

[19] Romero R, Miranda J, Kusanovic JP, Chaiworapongsa T, Chaemsaithong P, Martinez A, et al. Clinical chorioamnionitis at term I: microbiology of the amniotic cavity using cultivation and molecular techniques. J Perinat Med. 2015;43(1):19–36. 25720095 10.1515/jpm-2014-0249 PMC5881909

[20] Hanff PA, Rosol-Donoghue JA, Spiegel CA, Wilson KH, Moore LH. *Leptotrichia sanguinegens* sp. nov., a new agent of postpartum and neonatal bacteremia. Clin Infect Dis. 1995;20(Suppl 2):S237-239. 7548563 10.1093/clinids/20.supplement_2.s237

[21] De Martino SJ, Mahoudeau I, Brettes JP, Piemont Y, Monteil H, Jaulhac B. Peripartum bacteremias due to *Leptotrichia amnionii* and *Sneathia sanguinegens*, rare causes of fever during and after delivery. J Clin Microbiol. 2004;42(12):5940–3. 15583348 10.1128/JCM.42.12.5940-5943.2004 PMC535221

[22] Kotaskova I, Nemec P, Vanerkova M, Malisova B, Tejkalova R, Orban M, et al. First report of *Sneathia sanguinegens* together with *Mycoplasma hominis* in postpartum prosthetic valve infective endocarditis: a case report. BMC Infect Dis. 2017;17(1):563. 28806998 10.1186/s12879-017-2654-8 PMC5557263

[23] Shukla SK, Meier PR, Mitchell PD, Frank DN, Reed KD. *Leptotrichia amnionii* sp. nov., a novel bacterium isolated from the amniotic fluid of a woman after intrauterine fetal demise. J Clin Microbiol. 2002;40(9):3346–9. 12202577 10.1128/JCM.40.9.3346-3349.2002 PMC130742

[24] Boennelycke M, Christensen JJ, Arpi M, Krause S. *Leptotrichia amnionii* found in septic abortion in Denmark. Scand J Infect Dis. 2007;39(4):382–3. 17454911 10.1080/00365540601053022

[25] Manhart LE, Khosropour CM, Liu C, Gillespie CW, Depner K, Fiedler T, et al. Bacterial vaginosis-associated bacteria in men: association of *Leptotrichia*/*Sneathia* spp. with nongonococcal urethritis. Sex Transm Dis. 2013;40(12):944–9. 24220356 10.1097/OLQ.0000000000000054 PMC4188452

[26] Srinivasan S, Hoffman NG, Morgan MT, Matsen FA, Fiedler TL, Hall RW, et al. Bacterial communities in women with bacterial vaginosis: high resolution phylogenetic analyses reveal relationships of microbiota to clinical criteria. PLoS One. 2012;7(6):e37818. 22719852 10.1371/journal.pone.0037818 PMC3377712

[27] Peebles K, Velloza J, Balkus JE, McClelland RS, Barnabas RV. High global burden and costs of bacterial vaginosis: a systematic review and meta-analysis. Sex Transm Dis. 2019;46(5):304–11. 30624309 10.1097/OLQ.0000000000000972

[28] EisenbergT, Gronow S, Falgenhauer J, Imirzalioglu C, Muhldorfer K, Rau J, Blom J, Fawzy A, Glaeser SP, Kampfer P. Sneathia vaginalis sp. nov. (Fusobacteriales, Leptotrichiaceae) as a replacement of the species 'Sneathia amnii' Harwich et al. 2012 and 'Leptotrichia amnionii' Shukla et al. 2002, and emended description of Sneathia Collins et al. 2001. Int J Syst Evol Microbiol. 2019;71(3). 10.1099/ijsem.0.004663. 10.1099/ijsem.0.004663 33616512

[29] Collins MD, Hoyles L, Tornqvist E, von Essen R, Falsen E. Characterization of some strains from human clinical sources which resemble “*Leptotrichia sanguinegens*”: description of *Sneathia sanguinegens* sp. nov., gen. nov. Syst Appl Microbiol. 2001;24(3):358–61. 11822670 10.1078/0723-2020-00047

[30] Harwich MD Jr., Serrano MG, Fettweis JM, Alves JM, Reimers MA, Vaginal Microbiome C, et al. Genomic sequence analysis and characterization of *Sneathia amnii* sp. nov. BMC Genomics. 2012;13(Suppl 8):S4. 23281612 10.1186/1471-2164-13-S8-S4 PMC3535699

[31] Łaniewski P, Herbst-Kralovetz MM. Bacterial vaginosis and health-associated bacteria modulate the immunometabolic landscape in 3D model of human cervix. NPJ Biofilms Microbiomes. 2021;7(1):88. 34903740 10.1038/s41522-021-00259-8 PMC8669023

[32] Bolger AM, Lohse M, Usadel B. Trimmomatic: a flexible trimmer for Illumina sequence data. Bioinformatics. 2014;30(15):2114–20. 24695404 10.1093/bioinformatics/btu170 PMC4103590

[33] Wood DE, Lu J, Langmead B. Improved metagenomic analysis with Kraken 2. Genome Biol. 2019;20(1):257. 31779668 10.1186/s13059-019-1891-0 PMC6883579

[34] Wick RR, Judd LM, Gorrie CL, Holt KE. Unicycler: resolving bacterial genome assemblies from short and long sequencing reads. PLoS Comput Biol. 2017;13(6):e1005595. 28594827 10.1371/journal.pcbi.1005595 PMC5481147

[35] Parks DH, Imelfort M, Skennerton CT, Hugenholtz P, Tyson GW. CheckM: assessing the quality of microbial genomes recovered from isolates, single cells, and metagenomes. Genome Res. 2015;25(7):1043–55. 25977477 10.1101/gr.186072.114 PMC4484387

[36] Mikheenko A, Saveliev V, Hirsch P, Gurevich A. WebQUAST: online evaluation of genome assemblies. Nucleic Acids Res. 2023;51(W1):W601–6. 37194696 10.1093/nar/gkad406 PMC10320133

[37] Brettin T, Davis JJ, Disz T, Edwards RA, Gerdes S, Olsen GJ, et al. RASTtk: a modular and extensible implementation of the RAST algorithm for building custom annotation pipelines and annotating batches of genomes. Sci Rep. 2015;5:8365. 25666585 10.1038/srep08365 PMC4322359

[38] Olson RD, Assaf R, Brettin T, Conrad N, Cucinell C, Davis JJ, et al. Introducing the bacterial and viral bioinformatics resource center (BV-BRC): a resource combining PATRIC, IRD and ViPR. Nucleic Acids Res. 2023;51(D1):D678–89. 36350631 10.1093/nar/gkac1003 PMC9825582

[39] Jain C, Rodriguez RL, Phillippy AM, Konstantinidis KT, Aluru S. High throughput ANI analysis of 90K prokaryotic genomes reveals clear species boundaries. Nat Commun. 2018;9(1):5114. 30504855 10.1038/s41467-018-07641-9 PMC6269478

[40] Rodriguez-R LM, Konstantinidis KT. The enveomics collection: a toolbox for specialized analyses of microbial genomes and metagenomes. PeerJ Preprints. 2016;4:e1900v1901.

[41] Eisenberg T, Glaeser SP, Ewers C, Semmler T, Drescher B, Kampfer P. *Caviibacter abscessus* gen. nov., sp. nov., a member of the family Leptotrichiaceae isolated from guinea pigs (*Cavia porcellus*). Int J Syst Evol Microbiol. 2016;66(4):1652–9. 26813893 10.1099/ijsem.0.000922

[42] Jenkins DJ, Woolston BM, Hood-Pishchany MI, Pelayo P, Konopaski AN, Quinn Peters M, et al. Bacterial amylases enable glycogen degradation by the vaginal microbiome. Nat Microbiol. 2023;8(9):1641–52. 37563289 10.1038/s41564-023-01447-2 PMC10465358

[43] Srinivasan S, Morgan MT, Fiedler TL, Djukovic D, Hoffman NG, Raftery D, Marrazzo JM, Fredricks DN. Metabolic signatures of bacterial vaginosis. MBio. 2015;6(2):e00204–15. 10.1128/mBio.00204-15 PMC4453549 25873373

[44] Meng L, Meng M, Zhang R, Wubulikasimu A, Peng H, Zhang L, et al. Microbiome-producing SCFAs are associated with preterm birth via trophoblast function modulation. MBio. 2024;15(12):e0270224. 39526775 10.1128/mbio.02702-24 PMC11633107

[45] Schwecht I, Nazli A, Gill B, Kaushic C. Lactic acid enhances vaginal epithelial barrier integrity and ameliorates inflammatory effects of dysbiotic short chain fatty acids and HIV-1. Sci Rep. 2023;13(1):20065. 37973920 10.1038/s41598-023-47172-y PMC10654711

[46] Patra T, Koley H, Ramamurthy T, Ghose AC, Nandy RK. The Entner-Doudoroff pathway is obligatory for gluconate utilization and contributes to the pathogenicity of *Vibrio cholerae*. J Bacteriol. 2012;194(13):3377–85. 22544275 10.1128/JB.06379-11 PMC3434740

[47] Brothwell JA, Fortney KR, Batteiger T, Katz BP, Spinola SM. Dispensability of ascorbic acid uptake and utilization encoded by ulaABCD for the virulence of *Haemophilus ducreyi* in humans. J Infect Dis. 2023;227(3):317–21. 35876728 10.1093/infdis/jiac314 PMC10169391

[48] Ezraty B, Aussel L, Barras F. Methionine sulfoxide reductases in prokaryotes. Biochim Biophys Acta. 2005;1703(2):221–9. 15680230 10.1016/j.bbapap.2004.08.017

[49] Grosshennig S, Ischebeck T, Gibhardt J, Busse J, Feussner I, Stulke J. Hydrogen sulfide is a novel potential virulence factor of *Mycoplasma pneumoniae*: Characterization of the unusual cysteine desulfurase/desulfhydrase HapE. Mol Microbiol. 2016;100(1):42–54. 26711628 10.1111/mmi.13300

[50] Stephen AS, Millhouse E, Sherry L, Aduse-Opoku J, Culshaw S, Ramage G, et al. In vitro effect of *Porphyromonas gingivalis* methionine gamma lyase on biofilm composition and oral inflammatory response. PLoS One. 2016;11(12):e0169157. 28033374 10.1371/journal.pone.0169157 PMC5199072

[51] Gentile GL, Rupert AS, Carrasco LI, Garcia EM, Kumar NG, Walsh SW, et al. Identification of a cytopathogenic toxin from *Sneathia amnii*. J Bacteriol. 2020;202(13):e00162-e120. 32291280 10.1128/JB.00162-20 PMC7283592

[52] Ray RM, Bridy PV, Musicant AG, Chandravel S, Aziz YY, Cruz JC, Jefferson KK. Targeted deletion of the cytopathogenic toxin a gene in sneathia vaginalis. Mol Microbiol. 2025;124(6):481–90. 10.1111/mmi.70024 PMC12675970 40985292

